# Modern Sociology

**Published:** 1900-09-15

**Authors:** 


### Modern Sociology.

### WHAT THE NATIONS DRINK.

One of the earliest "free traders" was King Edward III. That energetic monarch, who in many respects deserves the title that has been given him of "Father of English commerce," determined to give his people plenty, if not peace, and to that end he took the duty off wine. If Edward III. had been fortunate enough to possess the statistics collected by Mr. H. Bence-Jones, and embodied by him in a paper he read before the Statistical Society, he would have seen that to admit wine into the country duty free was a very small benefit to the mass of his subjects. For while almost all people indulge in some form of intoxicant, the majority drink the liquor that their own country produces. The nations of Southern Europe drink wine, even the peasant has his flask of thin and rather sour ordinaire; those of the north drink beer almost as universally, though in the bleakest regions the preference is for more ardent forms of alcohol. But everywhere it is the drink that can be produced at home that is the favourite drink of the people.

The amount of wine that is drunk per head in wine-consuming countries is amazing. In France the consumption *per capita* is 24 gallons a year, while the average Italian drinks 20 gallons annually and the average Spaniard 18. But in mere quantity the beer-drinkers far exceed the wine-drinkers. Thus, the annual consumption of the United Kingdom is nearly 32 gallons per head; that of Germany is 27 gallons; that of the United States, where the Saxon element predominates, is 13 gallons. The general impression is that Germany is the country *par excellence* of beer-drinkers, but this is true only of parts of the Empire. Bavaria leads the world in the consumption of beer, its inhabitants drinking it at the rate of 56 gallons per head a year. Wurtemburg makes a good second with 43 gallons per head, while each inhabitant of the Grand Duchy of Baden may be reckoned as drinking 36 gallons in a year. But this indulgence is balanced by a very moderate rate of indulgence in other parts. Taken as a whole, Belgium leads the world in the matter of beer-drinking, its rate per inhabitant being 45 gallons. In fact Belgium seems to be a self-indulgent little country, for, in addition to this large amount of beer, its inhabitants drink, *per capita*, twice as much wine and spirits as the English do. Belgium drinks more of alcohol in every form than any other nation of Europe (of which statistics are to be had) with the possible exception of Denmark. Denmark is not so much of a beer drinking country, though its 20 gallons per head annually is a pretty large average; but it has the pre-eminence—affix to it what adjective you like—of drinking more spirits than any other nation—no less than three gallons per head. No other nation consumes more than two gallons a head. The countries which take even so much are France, Germany, Austria, Holland, Belgium, and Sweden. In the United Kingdom, the Russian Empire, the United States, and Switzerland the average consumption is about a gallon per inhabitant. Of course the spirit thus consumed is for the most part manufactured in the country where it is drunk. How little liquor is imported may be understood from the fact that only a little more than 4 per cent. of the sum which the national revenue of Great Britain derives from the taxation of alcoholic beverages comes from imported spirits.

A very interesting part of Mr. Bence-Jones's paper deals with the consumption of intoxicants in our colonies. It may be said in general terms that our children among the nations are much more temperate than we are ourselves. Australia is the most self-indulgent, and there the people drink approximately 1 gallon of wine, 10 gallons of beer, and three-quarters of a gallon of spirit per head annually. The wine is home-grown, which may account for the consumption of it being twice as great as that of Britain, for everywhere imported articles are the luxuries of richer classes. Australia is unique among the nations, however, for the large proportion of spirits which she imports, no less than 80 per cent. of her consumption of this form of alcohol coming from abroad, chiefly from our own country. There is, indeed, very little manufacture of spirits in Australia, nor does it seem to be increasing. It is very difficult to get reliable statistics of the consumption of the various intoxicants at Cape Colony, the official information obtainable being scanty. From four to six gallons of wine is produced annually, but as a great part of this (it is believed about two-fifths) is made into brandy, it is impossible to estimate with any exactness the amount that is drunk. There used to be a considerable export of Cape wine to Europe, and about 70,000 gallons are still sent abroad, but the greater part of the wine made in the Colony is now sent up to the Transvaal and other inland parts. The consumption of beer is very small, something under two gallons a year per head; nor is it increasing. Spirits are consumed at the rate of about one gallon per head, but it is not known how much of this is genuine brandy, and how much is the mysterious and unwholesome compound called "Cape-smoke."

Of all our colonies, Canada has the pre-eminence of being the most sober; indeed, it may be doubted if a more abstemious nation exists than the Canadian. Moreover, the consumption of alcohol is steadily decreasing. Spirits especially are falling into disfavour. In 1890 the Canadians consumed spirits at the rate of nearly a gallon a head; in 1898 the consumption *per capita* had fallen to little over half a gallon. Of wine, not more than a tenth of a gallon per head is drunk. Of beer the consumption is only three and a half gallons per head annually. The satisfaction with which temperance enthusiasts will regard this state of affairs may be tempered by the way in which they regard the increasing consumption of coffee both in Canada and in the United States, where also the consumption of spirits is diminishing. In Canada, while the consumption of spirits has fallen by nearly one half since 1890, the consumption of coffee has in the same period nearly doubled, though the absolute amount, 0·97 of a lb. per head, cannot be considered large. In the States the consumption of spirits was in 1890, 1·17 of a gallon per head; in 1898 it had sunk to 0·92. But during that time the consumption of coffee had risen from 8 lb. per head to 11 lb. The Americans of the United States drink far more coffee than any other people in the world. The Germans drink only 5 lb. per head, and the French only 3½.

